# COVID-19-related global health governance and population health priorities for health equity in G20 countries: a scoping review

**DOI:** 10.1186/s12939-023-02045-8

**Published:** 2023-11-03

**Authors:** Muriel Mac-Seing, Meron Gidey, Erica Di Ruggiero

**Affiliations:** 1 Department of Social and Preventive Medicine, School of Public Health, Université de Montréal, Québec, Canada; 2 Social and Behavioural Health Sciences Division & Centre for Global Health, Dalla Lana School of Public Health, University of Toronto, Ontario, Canada; 3 Centre de recherche en santé publique, Université de Montréal, Montréal, Canada; 4 Present Address: Social and Behavioural Health Sciences Division, Dalla Lana School of Public Health, University of Toronto, Toronto, ON Canada; 5 Social and Behavioural Health Sciences Division, Institute of Health Policy, Management and Evaluation & Centre for Global Health, Dalla Lana School of Public Health, University of Toronto, Toronto, ON Canada

**Keywords:** COVID-19, Equity, Global health governance, Population and public health priorities, COVID-19 consequences, G20 countries

## Abstract

Since the declaration of the COVID-19 pandemic, the promotion of health equity including the health of various population sub-groups has been compromised, human rights jeopardised, and social inequities further exacerbated. Citizens worldwide, including in the Group of 20 (G20) countries, were affected by both global health governance (GHG) processes and decisions and public health measures taken by governments to respond to COVID-19. While it is critical to swiftly respond to COVID-19, little is known about how and to what extent the GHG is affecting population health priorities for health equity in global economies such as the G20 countries. This scoping review synthesised and identified knowledge gaps on how the COVID-19-related GHG is affecting population health priorities for policy, programme, and research in G20 countries. We followed the five-stage scoping review methodology promoted by Arksey and O’Malley and the PRISMA Extension for Scoping Reviews guidelines. We searched four bibliographic databases for references conducted in G20 countries and regions and published in English and French, between January 2020 and April 2023. Out of 4,625 references and after two phases of screening, 14 studies met the inclusion criteria. G20 countries included in the review were Australia, Brazil, Canada, China, France, India, Italy, Japan, Russia, South Africa, the United Kingdom, the United States of America, and the European Union. We found insufficient collaboration and coordination and misalignment among governance actors at multiple levels. In most cases, equity considerations were not prioritised while unequal consequences of COVID-19 public health measures on population groups were widely reported. COVID-19-related population health priorities mainly focused on upstream and midstream determinants of health. Our scoping review showed the stark inequities of COVID-19 public health outcomes, coupled with a prevalent lack of coherent collaboration and coordination among governance actors. Moreover, governance as an object of empirical study is still emerging when examining its intersection with global health and population health policy, programme, and research. An urgent shift is required to effectively act upon structural health determinants that include transformative and comprehensive policies for prevention, equity, resilience, and sustainable health.

**Supplementary Information:**

The online version contains supplementary material available at 10.1186/s12939-023-02045-8.

## Introduction

The COVID-19 pandemic has exposed several major challenges in global health governance (GHG) highlighting competing priorities in protecting the health of populations and promoting and maintaining economic activity [1]. Global health governance is defined as “formal and informal institutions, rules, and processes by states, intergovernmental organizations, and nonstate actors to deal with challenges to health that require cross-border collective action to address effectively” [2]. In the context of our study, it was further defined as “governance arrangements needed to further agreed global health goals” that include health equity and social justice [3]. In this definition of GHG, equity considerations refer to the “ poor, vulnerable and disadvantaged” population groups who are affected differently by governance arrangements [3]. In early 2020, caught amid the tensions between two world economic and political powers, the United States of America (USA) and China, the World Health Organization (WHO) was judged to be too cautious and too slow to declare a Public Health Emergency of International Concern (PHEIC) to control the rapid spread of SARS-CoV-2, causing worldwide the coronavirus disease of 2019 (COVID-19) pandemic [4]. National governments and regional bodies, such as the European Union (EU) and the Association of Southeast Asian Nations [5], failed to effectively coordinate COVID-19 public health mitigation measures such as travel protocols, testing strategies, physical distancing approaches, data standards and reporting systems, and advice to the public [4].

Citizens in low-, middle-, and high-income countries, including those in the Group of 20 (G20), were affected by both GHG processes, decisions, and related guidance, in particular those promoted by WHO [6], and public health measures taken by governments to respond to COVID-19 [4, 7]. As a result, the promotion of health equity including the health of various population sub-groups was compromised, human rights jeopardised, and social inequities further exacerbated [8]. Evidence suggests that the COVID-19 pandemic disproportionately affected populations living and working in conditions of marginalisation or vulnerability such as frontline workers [9, 10], the elderly [11, 12], people of colour [13, 14], women [15–17], children [18, 19], incarcerated people [20], unhoused people [21, 22], Indigenous Peoples [14, 23], sexual minorities [24, 25], and people with disabilities [26, 27]. In most countries, equity was insufficiently considered in the design of these measures and might have led to several socioeconomic consequences [28]. Literature reported the gendered impacts of COVID-19 public health measures that were most experienced by women working in precarious jobs while continuing to take care of household chores and attending to childcare and children’s home-based school needs and elderly family members [15]. However, despite difficult access to health and social care for marginalised migrants due to COVID-19 and systemic discrimination, their access was facilitated through the coordinated work of civil society organisations in three European countries [29].

Moreover, the pandemic response in the first year of COVID-19 led to the ‘covidization’ of health research agendas and impact their pursuit for health equity, where financial, human, and technical resources were massively channelled to respond to COVID-19 [30]. Priorities for current and future population health research such as health promotion and prevention of both communicable and non-communicable diseases with equity as a central tenet continue to be at risk of being neglected due to the pandemic. While it is critical to swiftly respond to COVID-19, little is known about how and to what extent the GHG is affecting population health priorities for health equity in global economies such as the G20 countries. Hence, this scoping review aimed to examine the existing academic literature on COVID-19-related GHG and its impact on population health priorities (research, programme, and policy) in G20 countries. Specifically, we asked the following questions. (1) What are the COVID-19-related GHG features in G20 countries? (2) What are the COVID-19-related GHG consequences on population health priorities and equity issues in G20 countries? (3) Which marginalised or vulnerable populations are affected by the COVID-19-related GHG decisions in G20 countries? (4) What are the population health priorities (policy, programme, and research) and gaps in G20 countries?

## Methods

**Fig. 1:**
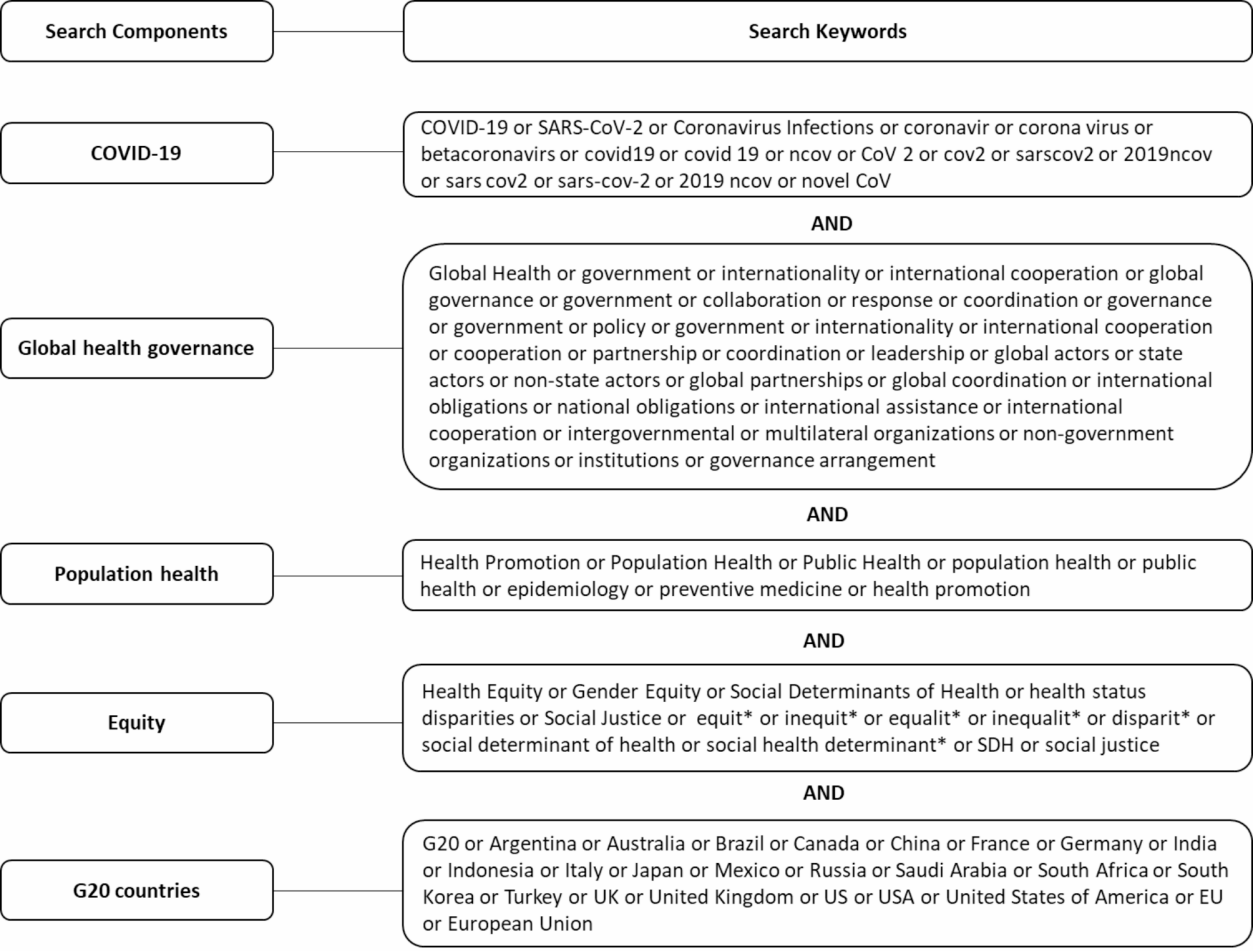
Search terms

This scoping review is embedded in a larger programme of work that also includes a qualitative multilevel study to examine the relationships between COVID-19-related GHG and population health research priorities in Canada. The scoping review protocol was registered in Open Science Framework [31]. We identified peer-reviewed literature which focused on five concepts: COVID-19, GHG, population health, equity, and the G20 countries. We followed Arksey’s & O’Malley’s five-stage scoping review framework [32] and PRISMA Extension for Scoping Reviews (PRISMA-ScR) Checklist [33]. We used the Population, Concept, and Context (PCC) framework for scoping reviews [34]. Figure 1 details the search terms and keywords we used.

### Information sources and search strategy

We developed the search strategy in consultation with the public health-related librarian at the University of Toronto. We searched Medline, Global Health, EMBASE, and Web of Science. Search terms and keywords were initially tested in Medline, and subsequently applied to the other bibliographic databases.

### Study eligibility

We included original research, reviews, and commentaries in peer-reviewed literature in the review if they fulfilled the following eligibility criteria: 1) reported in English or French and published from January 2020 to April 2023, in at least one or more of the G20 countries, and address the following concepts of COVID-19, equity, GHG (implicitly or explicitly discussed with related terms such as global partnership, cooperation and coordination, actors (e.g., global, state, non-state), intergovernmental organisations, international or national obligations, and national priorities), and population health priorities (policy, programmes, and research). We excluded references that were letters to the editor, editorials, perspectives, conference proceedings, opinions, and interviews, or solely addressed GHG for a specific health issue/disease/condition that was unrelated to COVID-19 or examined population health priorities outside of G20 countries or did not address vulnerable/marginalised groups or equity issues.

### Study selection

Two reviewers (MMS and MG) independently assessed academic literature search results against the eligibility criteria in two phases: (1) titles and abstracts and (2) full texts. Discrepancies were resolved through discussions with the third reviewer (EDR) and the criteria were fine-tuned. A pilot test of title and abstract screening was completed for the first 50 search results. A flowchart was produced to document the screening and selection process and is provided in the results section.

### Data management, charting, and analysis

Results were imported and managed with Zotero (Version 5.0.96.3). The academic references were imported to COVIDENCE (https://www.covidence.org/) via the network of libraries of the University of Toronto where duplicate references were removed. For studies at the eligibility phase that met inclusion criteria, data charting was facilitated using a standardised table in Excel for the following aspects: authors’ names, publication year, place of study, study objectives, implicit or explicit GHG features, equity considerations, determinants of health (upstream, midstream, downstream), population groups in conditions of vulnerability or marginalisation, main results, and implications for population health priorities (policy, programme, research). Data was synthesised narratively. We adopted a descriptive analysis informed by our specific scoping review research questions.

## Results

### Characteristics of studies and reports included

**Fig. 2:**
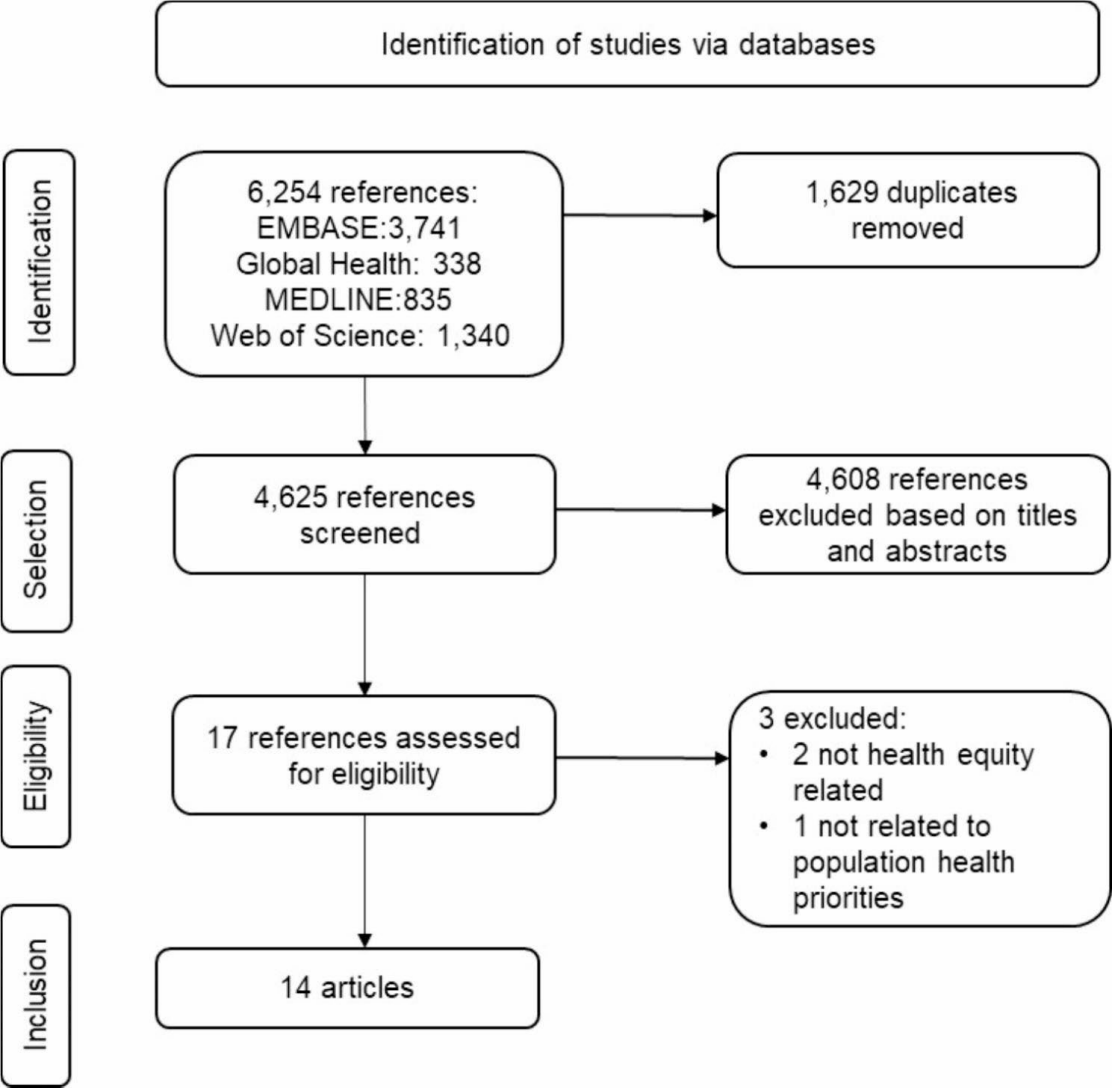
Flow chart

Out of 6,254 references identified, 1,629 duplicates were removed through COVIDENCE. Among 4,625 screened titles and abstracts, 4,608 were excluded because they did not meet the inclusion criteria. Seventeen full-text papers were then assessed for their eligibility, of which three were excluded as they did not meet the inclusion criteria (Fig. 2). In the last phase, 14 studies were included. Thirteen G20 countries and regions included in the reviewed references were Australia, Brazil, Canada, China, France, India, Italy, Japan, Russia, South Africa, the United Kingdom (UK), the USA, and the EU region.

Most objectives of included peer-reviewed studies addressed the impacts of lockdowns, stay-home directives, and other public health control measures on different populations living and working in vulnerable conditions (e.g., people who are socioeconomically disadvantaged, women in situations of abuse, and migrants) [35–48]. Two specifically examined the gendered effects of COVID-19 measures on women in South Africa [40] and Japan, China, Singapore, and Italy [46]. One study looked at the relationships between the effects of fiscal stimulus and COVID-19-related economic consequences in Japan [41]. Two other studies examined the cross-border effects and challenges of public health measures between the Republic of Ireland and Northern Ireland (UK) [45], and Canada and the USA [39]. Three studies examined the COVID-19-related public policy consequences in two regions, the EU [38, 42] and BRICS (Brazil, Russia, India, China, and South Africa) [44] (Table 1).

**Table 1:** Characteristics of literature included

Type of Literature	COVID-19-related GHG features	Focus on Equity	Determinants of health (upstream, midstream, downstream)	Differential equity consequences of COVID-19 measures	Population health priorities (policy, programme, research)
Agoramoorthy (2021): India (1)	Lack of political coordination and collaboration between the government and health experts.	Implicitly addressed with reference to economic and political inequality (e.g., Indian caste system).	Mostly downstream: income, job and food insecurity, and mental health. Some midstream: unstable, and inadequate housing quality.	Explicitly discussed with reference to marginalized groups such as Indigenous people, Dalits, and migrant worker living within lower social classes.	Policy priorities regarding political cross-collaboration and compassionate civil liberty disaster management policies, epidemic control strategies that concern the elderly.
Alrob (2022): Canada (2)	Implicitly addressed: discrepancy between what the UN and WHO recommended for the health and wellbeing of migrants, refugees, and asylum seekers).	Explicitly addressed through structural health inequalities created by the Canadian government among different types of migrants.	Mostly upstream through COVID-19-related mobility policies for migrants.	Explicitly discussed with reference to groups of migrants (seasonal workers, refugees, and asylum seekers) who are affected differently due to policies adopted favouring seasonal workers and jeopardising the human rights of other groups of migrants.	COVID-19-related policies should consider the human rights of migrants.
Bajos (2021): France (3)	Lack of political coordination and collaboration discussed.	Explicitly discussed social inequities: living and working conditions for working class individuals, highest COVID-19 related mortality rate amongst the working class.	Upstream: preventative policies. Some midstream: living and working conditions.	Implicitly discussed disadvantaged groups based on education and income but no mention of people with multiple and other intersecting social disadvantages.	Explicitly addressed: call for upstream preventative policies.
Brooks (2022): European Union (4)	Regional governance and coordination on health through health, pharmaceutical, and fiscal policies	Explicitly discussed: inequalities within and between EU member states (e.g., in health systems and support to populations).	Upstream: health, market (pharmaceutical), and fiscal (recovery plans) policies. Midstream: quality of housing and education, and access to healthcare.	Explicitly discussed disadvantaged populations groups (e.g., workers and people living in densely populated housing and people with comorbidities at risk to contract COVID-19).	Policy implications and priorities to consider supporting the economic development and reducing at the same time inequalities between EU regions.
Combden (2021): Canada and the USA (5)	Implicitly discussed through COVID-19 pandemic responses throughout world and impacts on how national authorities and governments responded (e.g., lack of leadership reported in the USA).	Explicitly discussed through racial/ethnic social and health inequities regarding COVID-19 consequences, lack of data collection on race/ethnicity, and history of racism in healthcare systems.	Upstream: racism. Midstream: access to healthcare and personal protective equipment (PPE). Downstream: employment, age (especially in the USA) and individual comorbidities (e.g., diabetes and obesity).	Explicitly discussed with reference to young and older people, people with comorbidities being more at risk of COVID-19, people living in long-term care, LGBTQ + communities and racialized people.	Programmatic priorities to have for example a robust public health surveillance system, rapid accessible mass testing, rapid collection, collection, analysis and dissemination of testing data, and clear and consistent communication from government and public health leaders.
Dekel (2021): South Africa (6)	Implicitly addressed: no consideration of gender equity in national COVID-19 measures resulting in GBV shadow pandemic.	Implicitly discussed gender inequalities: increased intimate partner violence during COVID-19 strict lockdown period.	No clear consideration of determinants of health.	Explicitly addressed with reference to gender inequities experienced among South African women in shelters of three provinces.	Implicitly discussed programmes: diversify channels of communication and support to abused women and policies (e.g. gender equity integrated national pandemic policies).
DeWit (2020): Japan (7)	Explicitly addressed: COVID-19 countermeasures bridging national and international imperatives.	Explicitly discussed systemic risks (e.g., climate change), and the risk of increased inequality if long-term sustainability is not acted upon in policies (e.g., strengthening social protections).	Implicitly approached upstream determinants of health (e.g., fiscal policies) and holistic policies.	Implicitly addressed with a brief and general reference to “the most disadvantaged” populations.	Call for strategies and policies that bolster long-term sustainability and resilience.
European Centre for Disease Prevention and Control (2020): European Union and the UK (8)	Lack of coordination and collaboration between national/regional authorities and civil society organisations discussed.	Implicitly discussed different conventions: Charters of Fundamental rights of the EU and Convention on the rights of persons with disabilities.	Implicitly discussed midstream and downstream social determinants of health: housing, education, occupation, disability, access to care and protective personal equipment.	Explicitly discussed diverse communities: ethnic minorities, irregular migrants, LGBTQI people, people with diseases, people experiencing homelessness, people with disabilities, people using drugs.	Implicitly discussed: continuity of services, material supports, use of digital technologies, community engagement approach, and needs assessment and evaluation of services.
Griffiths (2022): Australia (9)	Addressed through WHO’s advice to use lockdowns for short periods versus what Australia decided to put in place, i.e. a 4-month long lockdown.	Implicitly addressed through the negative mental health consequences experienced by people who lost their job during lockdowns.	Downstream: engagement in work and social interaction among people experiencing loss of work during the pandemic.	Implicitly discussed the consequences the mental health of youth and older people in the State of Victoria.	Programme-related: priority to consider people’s engagement in work and their mental health.
Jiao (2022): Brazil, Russia, India, China, South Africa (10)	Explicitly addressed through BRICS regional cooperation related to COVID-19 responses (health, vaccination, and financial support).	Explicitly addressed through national support to cope with COVID-19 (e.g., financial support for COVID-19 treatment in China or single moms in South Africa).	Upstream: regional governance to respond to COVID-19.	People living in poverty and disadvantaged conditions such as in slums in India and Brazil, refugees, and migrants in South Africa.	Policy and programmatic priorities to focus on regional cooperation and health equity.
O’Connor (2021): The UK and Republic of Ireland (11)	Lack of political coordination and collaboration of public health measures along the ROI-NI border explicitly discussed.	Implicitly addressed through differences in reported COVID-19 cases and mortality rates due to cross-border policy disparities.	Implicitly approached midstream social determinants of health: access to jobs, healthcare services and schools.	Based on people of different age groups, parents of children across the border, and people who had COVID-19.	Recommendation for a ‘all-island’ approach in responding to the pandemic considering the specificities of populations on the two sides of the border.
Smith (2021): China, Hong Kong, Canada, and the UK (12)	Implicitly addressed: Case study outbreaks and responses discussed were mostly generic, one-sized-fits- all but some examples of efforts grounded in intersectional approaches.	Explicitly addressed gender equity and feminist intersectional approach.	Implicitly approached downstream and midstream determinants: race/ethnicity, age, disability, gender.	Explicitly discussed intersectional consequences of pandemic responses on women and people living at the intersection of multiple inequities (e.g., essential workers).	Implicitly discussed: call for integration of a feminist intersectional approach in pandemic responses, government policies and research, and outlines policy gaps.
Szylovec (2021): Brazil (13)	Lack of political coordination and collaboration discussed.	Explicitly discussed social and economic inequality.	Implicitly approached midstream determinants: socially vulnerable people in poor living conditions, elderly people, workers in the informal sectors.	No technical recommendations from the Ministry of Health were provided to people living in poor living conditions (e.g., favelas and those without access to water, and Indigenous Brazilian communities).	Recommendations to include vulnerable populations from the start when designing emergency measures, need for unified leadership during health crises, using health equity approach for future research.
Wang (2021): Japan, Italy, China, and Singapore (14)	Implicitly addressed: Briefly mentioned how the results will help policymakers explore effective response measures within the capabilities of their jurisdiction.	Explicitly discussed through the inequities experienced by socio-economically disadvantaged groups.	Discussed midstream and downstream determinants: socioeconomic factors, age, living and working conditions.	Implicitly discussed: socio-economically disadvantaged and the elderly.	Implicitly addressed: recommend countries choose response strategies based on their local specificities.

In the next sections, we report four main themes: (1) insufficient coordination and misalignment among governance actors at multiple levels as mostly discussed GHG features, (2) equity considerations, (3) consequences of COVID-19 public health measures on population groups, and (4) COVID-19-related population health priorities.

### Insufficient coordination and misalignment among governance actors at multiple levels

Despite publicly available COVID-19 public health guidance from WHO to Member States and public health authorities to facilitate enhanced coordinated efforts worldwide [6], most studies and reports included in the review reported a lack of [35, 37, 42, 45, 47] or implicit [40, 46, 48] coordination among international, national, and regional leaders and health experts. Among articles that reported strikingly insufficient coordination among political and health governance actors, one study examined the COVID-19-related political and public health decisions between Northern Ireland (in the UK) and the Republic of Ireland [45]. The study reported divergent COVID-19 public health measures for both populations who share a common border on the island despite cross-border interdependent activities for travel, employment, and healthcare [45]. Another study conducted in Brazil reported important COVID-19 social and political disruptions generated by misalignment between what the Ministry of Health recommended (based on WHO’s guidance) and what intra-federal states decided to implement and what the Brazilian President conveyed by downplaying COVID-19 as a “little cold” [47]. A study conducted in Japan explicitly discussed COVID-19-related fiscal policy and interlevel coordination (international, national, and sub-national) along with the 2030 Agenda’s Sendai Framework on Disaster Risk Reduction (SFDRR) through a National Resilience Plan [41]. However, two studies addressed a regional effort to coordinate health, pharmaceutical, and financial policies to respond to COVID-19 in the EU [38] and health, vaccine, and economic cooperation in the BRICS countries [44].

### Equity considerations

While five out of 14 studies implicitly addressed equity considerations [35, 40, 42, 43, 45], nine explicitly addressed equity regarding socioeconomic [37, 39, 47], financial [44], gender [46], and health [36, 38, 44, 48] inequities, and climate resilience [41]. A comparative study of the policies adopted in China, Hong Kong, Canada, and the UK showed that women faced multiple concomitant burdens in employment, housework, childcare, and care of other family members due to COVID-19-related public health measures when compared to their male counterparts [46]. Another study conducted in France found people living in economically precarious conditions were more at risk of COVID-19 than those who were economically more privileged [37]. Among the studies which explicitly addressed equity considerations, they also discussed more upstream determinants of health in terms of regional cooperation and collaboration [38, 44], preventative policies to reduce social inequities [37], fiscal [38, 41, 44], and climate change policies [41], intersectional discriminations in society (e.g., racism and sexism) [39, 46], and unfavorable social policies leading to poor living conditions that increase the risks to COVID-19 [47, 48]. Studies that implicitly addressed equity reflected determinants of health more at the midstream and downstream levels that included income [35], mental health [35], housing [42], education [42], and age [47].

### Consequences of COVID-19 public health measures on population groups

The main population and public health measures to control COVID-19 in G20 countries in studies included ranged from social and fiscal measures [38, 40, 41, 44–46, 48] to non-pharmaceutical interventions (NPI) such as physical distancing and stay-at-home instructions [35, 36, 39, 40, 42, 44, 45, 47, 48], and lockdowns, as a larger-scale blocking NPI measure [35, 37, 43, 44, 46, 48]. Pharmaceutical interventions (e.g., vaccines) [35, 38, 44] and COVID-19 testing [39, 42, 44, 46] were also addressed (Table 2). These measures affected different population groups. One paper conducted in the EU and the UK was the most comprehensive. It addressed the different population groups living and/or working in conditions of vulnerability or marginalisation that included women, older adults and youth, precarious workers, homeless people, people with disabilities, Black and other People of Colour, LGBTQ + communities, and others (e.g., people using drugs, sex workers, and people who are at an elevated risk of contracting COVID-19 due to comorbidities) [42]. Two studies specifically examined the consequences of measures to control COVID-19 on women in South Africa [40] and China, Hong Kong, Canada, and the UK [46]. South African women survivors of intimate partner violence living in shelters during a lockdown reported the double challenge they were experiencing, with some of them sharing the following: “I will rather be killed by corona than by him…” illustrating the ongoing ‘shadow’ gender-based violence epidemic in South Africa [40]. In the other study that adopted an intersectional feminist lens, study findings showed that women in the four countries examined faced additional childcare responsibilities at home, were less protected financially and socially, and were at higher risk of exposure to COVID-19 given the type of employment they had (e.g., frontline, and domestic work) [46]. On the other spectrum, a 4-hour notification of an executive decision by the Indian Prime Minister to enforce a country-wide lockdown had severe negative consequences on millions of internal migrant workers and homeless people who faced hunger, increased vulnerability, marginalisation, poverty, jail time, and death [35]. Another population-based study on the mental and physical health consequences of a four-month lockdown on people living in the State of Victoria, Australia, found that they were worse off than people of other Australian states who did not experience such a lockdown [43].

**Table 2:** COVID-19 population and public health measures and populations groups affected

	COVID-19 population and public health measures*					Populations in conditions of vulnerability or marginalisation**								
	BM	PI	NPI	SM	COVID-19 testing	Women	IP	PW	Old or young people	BPOC	LGBTQ+	PEH	PWD	Other
Agoramoorthy (2021): India	X	X	X				X	X				X		
Alrob (2022): Canada			X					X						X
Bajos (2021): France	X							X						Disadvantaged groups
Brooks (2022): European Union		X		X				X						X
Combden (2021): Canada and the USA			X		X				X	X	X			X
Dekel (2021): South Africa	X		X	X		X				X				
DeWit (2020): Japan				X										Disadvantaged groups
European Centre for Disease Prevention and Control (2020): European Union and the UK			X		X	X		X	X	X	X	X	X	X
Griffiths (2022): Australia	X								X					X
Jiao (2022): Brazil, Russia, India, China, South Africa	X	X	X	X	X	X		X						X
O’Connor (2021): The UK Republic of Ireland			X	X	X				X					X
Smith (2021): China, Hong Kong, Canada, and the UK	X			X	X	X					X			
Szylovec (2021): Brazil			X		X				X					
Wang (2021): Japan, Italy, China, and Singapore	X		X	X	X			X	X					

*COVID-19 population and public health measures

BM: Blocking measure (e.g., lockdowns)

PI: Pharmaceutical interventions (e.g., vaccines)

NPI: Non-pharmaceutical interventions (e.g., physical distancing, travel restrictions, stay-at-home, quarantine)

SM: Social measures (e.g., closures of schools and businesses and fiscal stimulus)

COVID-19 testing

**Populations in conditions of vulnerability or marginalisation

Women

IP: Indigenous Peoples (e.g., First Nations, Inuit, and Metis People, and tribal communities in India)

PW: Precarious workers (e.g., migrant, healthcare, informal sector, socioeconomically disadvantaged people)

Young or old people

BPOC: Black and People of Colour

LGBTQI+: Lesbian, Gay, Bisexual, Transgender, Queer, Intersex + people

PEH: People experiencing homelessness

PWD: People with disabilities

Other: People using drugs, people who suffered from COVID-19 or are at elevated risk of severe COVID-19-related diseases, and other disadvantaged groups

### COVID-19-related population health priorities

The COVID-19-related population health priorities discussed in the included studies and reports are regrouped into two main categories: policy and programme. Policy-related priorities focused on upstream policy aspects that aim to transform the systems, such as in public health and the governance structures. Studies recommended that policy priorities should promote regional cooperation and coordination in the EU [38] and BRICS [44], the human rights of different groups of migrants in Canada [36], economic and fiscal policies in the EU [38], and preventative policies in France [37] to reduce and avoid future health inequities among the economically disadvantaged population. Moreover, an intersectional feminist lens in COVID-19 response policies in China, Hong Kong, Canada, and the UK [46], and gender equity-oriented policies in South Africa in pandemic responses for more integrated, inclusive, and equitable responses [40] were suggested. In Japan, longer-term macro level policies to foster sustainability and resilience (including climatic) [41] were considered future crucial population and public health priorities. In the case of Ireland, a more comprehensive ‘all island’ policy approach was encouraged for more coherent and greater synchronisation of the COVID-19 pandemic response to address the cross-border specificities faced by the populations across the two sides of the insular border [45]. In India, following the negative consequences of COVID-19 blocking measures on the domestic migrant populations, a ‘compassionate civil liberty component’ was recommended for integration into policy-making decisions to respond to national disasters such as COVID-19 and to avoid severe distress and preventable death among populations living and working in conditions of dire vulnerability [35]. Regarding programmatic priorities, these focused on midstream and downstream interventions that included community needs assessments and evaluation of services provided during COVID-19 in the EU [42], the inclusion of vulnerable populations from the onset of emergency measures development in Brazil [47], people’s engagement and mental health impacts from a lockdown in Australia [43], and consideration of country/local needs for tailored COVID-19 responses in Italy, Japan, China, and Singapore [48]. No studies included in the review reported any population health research priorities for future consideration.

## Discussion

This scoping review sought to synthesise and identify the gaps in the existing literature on COVID-19-related GHG and its impact on population health priorities including equity considerations in G20 countries. We report three main findings. The first finding addresses the GHG characteristics reported in included studies. The second finding highlights equity considerations and the population groups that were most affected by COVID-19 in G20 countries. The third finding reports the main population and public health priorities addressed in included studies. Equity cuts across all three main findings – it is one key principle of good governance [49], which operates at different levels of GHG [3].

First, although all studies addressed COVID-19 public and population health policies and measures adopted in the aftermath of important GHG policies and decisions promulgated by WHO for all countries to respond to COVID-19, explicit discussions between the intersection of COVID-19-related-GHG and population health priorities in G20 countries remains sparse. Coordination and cooperation as well as equity considerations were the main COVID-19-related GHG features addressed in included studies, whereas other key principles of governance such as accountability and strategic direction (vision of leaders and leadership approaches) have not been explicitly addressed [49]. It is argued that given the sudden and then evolving health crisis generated by COVID-19, many countries including those in G20 have been preoccupied to control the spread of COVID-19 domestically and protect the health of their own citizens and economies, contributing to limited coordination among different governance actors in the G20 countries. The reported insufficient collaboration and coordination among actors and decision-makers at different levels of health governance (global (e.g., WHO), regional (e.g., EU), national (e.g., country level), and sub-national (e.g., provinces/states) is a stark reflection of a fragmented aim toward global collaborative efforts to respond forcefully to the pandemic. For example, the COVAX initiative, spearheaded by WHO and other GHG actors, aimed to foster cooperation and coordination among multilevel GHG actors for vaccine equity. However, at the same time, national priorities focused on measures such as closing borders and banning citizens of other countries to enter one’s territory [50]. While there have been specific attempts to coordinate COVID-19-related public health and economic measures in the EU in 2020 and 2021, there was “no coordinated and effective” COVID-19 response among European member states. Instead, policy choices and responses were nationally driven [51], resulting in socioeconomic and health inequities faced by different population groups in situations of vulnerability and/or marginalisation.

Second, as our review findings suggest, equity considerations – a key principle of good governance [49] – and population groups living and working in conditions of vulnerability or marginalisation were, again, the victims of after-thought COVID-19 responses across the G20 countries. Two compelling examples from this scoping review highlight the disconnect between what WHO promoted to control the spread of COVID-19 and the sudden decision of the Indian government to implement a country-wide lockdown, which had severe consequences on groups of migrant workers and homeless people [35]. Similarly, there was a misalignment between the decision of the then Brazilian President and what the different sub-national lines of the ministry of health promoted, following WHO guidelines. This situation further jeopardised the health of Indigenous people and those living in favelas [47]. These findings are corroborated by a recent scoping review of 49 studies that examined GHG and health equity in the context of COVID-19 [8]. They reported that human rights and inequities were undervalued by key governance actors who took decisions to respond to COVID-19, coupled with structural factors (e.g., gender discrimination and racism) that facilitated the exacerbation of such inequities in low-, middle-, and high-income countries [8]. A recent study conducted in France that explored the social and health inequities in the COVID-19 response found that despite a strategic opportunity to address these inequities, it did not materialise mainly due to a dominant biomedical epidemiological framework adopted during the COVID-19 emergency phase that gave precedence to the virus rather than to the socio-structural determinants of health [52]. Nonetheless, the COVID-19 pandemic has mobilised international actors to address these inequities, in particular vaccine inequities, through the Access to COVID-19 Tools Accelerator initiative, which aims to expedite the “development, production, and equitable access to COVID-19 tests, treatments, and vaccines” globally [53]. Hassan and colleagues argue that one of the pandemic lessons learned to control COVID-19 is the political commitment of decision-makers to optimise population health and reduce economic loss through a constant equity lens [54]. Dalingwater further begs the question of whether GHG should be redesigned, and national public health systems significantly re-financed to better fit the purpose of tackling unjust and avoidable gender, social, and economic disparities [55] to reconnect COVID-19-related GHG to population health priorities at international, national and sub-national levels.

Third, we found that many of the studies included in the review highlighted COVID-19 population and public health priorities that concerned policy and programmatic priorities and implications that addressed upstream and midstream determinants of health for more equity considerations among population groups living and working in conditions of vulnerability or marginalisation. However, none of the included studies addressed research as a key priority for population health improvement [56]. Since the onset of the COVID-19 pandemic, academic literature has outlined the importance of linking equitable health outcomes for various population groups with structural determinants of health that have important bearings on population health, considering both global and national public health. Our scoping review findings corroborate the literature that reported the importance of promoting community approaches within health systems strengthening to mitigate the negative COVID-19 impacts on health, especially among population groups that are more vulnerable to these impacts such as women and girls, migrant workers, people with disabilities, elderly people, refugees and displaced people, and ethnic minorities [57]. Community-oriented approaches including community engagement and need assessments are better positioned to identify priorities that address the numerous determinants of health (e.g., racism and structural oppressions) to further health equity [58]. Our findings also showed that sub-optimal GHG characterised by insufficient and incoherent collaboration and coordination among global and public health actors can potentially lead to downstream inequities. It has been suggested that an urgent “paradigm shift” away from a COVID-19 biomedical lens toward a more integrative investment in health systems strengthening that includes addressing the prevention of non-communicable diseases (through upstream interventions) beyond the management of infectious diseases requires a synergetic overhaul of global solutions and local actions [59]. This shift further contributes to a macro-level thinking considering GHG as an important structural determinant of public and population health for equity health not only at different national levels but globally as well [60].

This study presents both strengths and limitations. A major strength of this study was its comprehensive approach, examining the intersection of three dimensions together and not separately, notably GHG, equity considerations, and population health priorities. This analysis allowed us to highlight major inequitable consequences for the health of various population groups in G20 countries and identify research gaps to inform future perspectives. However, only 14 studies were included in the last stage of the scoping review. The specific scoping review inclusion criteria examining the intersection between COVID-19-related GHG features and population health priorities in G20 countries might have contributed to this small number of included studies. Our inclusion criteria looked for multiple intersecting concepts, which might have limited the inclusion of studies that only examined one concept. Compounded on this, it is suggested that governance as an object of empirical study is still emerging. Studies included were conducted in 13 countries/regions out of the G20 countries (65%), leaving the perspectives of other G20 countries. However, the main thematic areas discussed in the scoping review corroborate the findings in the literature as previously described. Another limitation of this study was it only included studies published in English and French, which may have excluded the perspectives of studies published in other languages.

## Conclusion

Three years have passed since the onset of the COVID-19 pandemic, and we are yet to witness its exit and equitable control worldwide. Despite initial signals from the global community, starting with WHO’s call to respond collectively to COVID-19, nationalism rapidly took hold, and existing inequities affecting populations in conditions of vulnerability or marginalisation were exacerbated and persisted throughout COVID-19. Our scoping review showed the duress of inequitable COVID-19 public health outcomes following drastic global and national public health decisions that were taken to address COVID-19, coupled with limited cohesive and coherent collaboration and coordination among governance actors at global, national, and sub-national levels. Based on the COVID-19 population and public health priorities proposed in the review, key policy recommendations include an urgent shift in addressing upstream and midstream determinants of health. This includes transformative policies for prevention, resilience, sustainable health, and health equity, along with conducting comprehensive analyses of the intersection between GHG, equity considerations, and population health priorities. Another policy recommendation is for GHG actors at global, regional, national, and sub-national levels to systematically cooperate and collaborate with one another, even amid the urgency to control COVID-19 as illustrated in the studies conducted in the G20 countries. A lack of careful and accountable examination of and research on GHG decisions and equity considerations can lead to important consequences for vulnerable population groups as reported in this scoping review. These recommendations are not only crucial for addressing COVID-19, but also for any other crises such as climate change, that profoundly disrupt and challenge how we strategize, plan, and act on global health governance for equity. More than decades ago, Ilona Kickbusch addressed the ‘glocal’ (global + local) interrelationships between global challenges and priorities and local needs and action through healthy cities and a network of governing bodies collaboratively using a system-thinking approach that tackles health through integrated and multilevel strategies [61]. Given the backdrop of climate change and erosion in global and national solidarity, we are doomed to succeed or fail together in responding to COVID-19 and future pandemics.

### Electronic supplementary material

Below is the link to the electronic supplementary material.

Supplementary Material 1

## Data Availability

All secondary data are available from published studies of articles included in the scoping review.

## Abbreviations

BRICS: Brazil, Russia, India, China, and South Africa
EU: European Union
G20: Group of 20 countries
GHG: Global Health Governance
PHEIC: Public Health Emergency of International Concern
UK: The United Kingdom
USA: The United States of America
WHO: World Health Organization
